# Relationship Between Ways of Coping and Quality of Life in Married Women: Toward Mental Health Promotion

**DOI:** 10.5812/ircmj.12728

**Published:** 2013-08-05

**Authors:** Arash Mirabzadeh, Monir Baradaran Eftekhari, Ameneh Setareh Forouzan, Homeira Sajadi, Hassan Rafiee

**Affiliations:** 1 Social Determinant of Health Research Center, University of Social Welfare and Rehabilitation Science, Tehran, IR Iran; 2 Department of Psychiatry, University of Social Welfare and Rehabilitation Science, Tehran, IR Iran; 3 Undersecretary for Research and Technology, Ministry of Health and Medical Education, Tehran, IR Iran

**Keywords:** Quality of Life, Women, Mental Health

## Abstract

**Background:**

Adaptive ways of coping with stress are as a major component of mental health and also this is considered as a key element in quality of life.

**Objectives:**

The aim of this study was to investigate the relationship between quality of life and coping mechanisms in married women ages between 18-65 years in order to develop appropriate intervention programs to promote mental health.

**Patients and Methods:**

This study was a part of interventional project to mental health promotion in married women that completed through a cross sectional studies using two standard questionnaires: Ways of Coping (WOC) and Quality of Life questionnaire (WHO, QOL-BREF).

**Results:**

The most and the least used ways in coping with stress were Planful Problem Solving and Confronting Coping. Considering the quality of life, the most and the least scores were related to social dimension and mental health. Also women who have higher quality of life used more Positive Reappraisal way and less Escape-Avoidance way to deal with stress (P < 0.05).

**Conclusions:**

It seems that implementation of an appropriate interventional program related to adaptive ways of coping in order to deal with stress is effective in mental health and quality of life promotion.

## 1. Background

In 2006, for the first time, the Commission on the Social Determinants of Health (CSDH) analyzed the social causes of health inequities and emphasized that factors such as gender, occupation, education and psychological problems are effective in health discrimination (1). In unequal societies, chronic stress of struggling with material disadvantage is intensified and mental health is as a determinant to understanding the impact of inequalities on health and other outcomes (2). Based on WHO definition, mental health is a fundamental element to cope with adversity or stress to reach full potential and humanity. The study of stress and coping as a central focus of mental health promotion indicates the importance of these topics for psychological and physical well-being in community level (3).

According to the Folkman- Lazarus theory, the basic concept of coping is a dynamic interaction between person and environment to manage external or internal demands. Coping strategies are classified in two ways: problem-focused coping, which is actively or behaviorally altering the external person–environment relationship, and emotion-focused coping, which is altering the personal or internal meaning or relationships (4). Problem-focused coping ways involve efforts to change or eliminate the source of stress, whereas emotion-focused coping strategies tend to regulate the negative emotional consequences of the stressors. Unfortunately women prefer deal with stress via emotion-focused coping strategies that increase the daily stress (5).

Confronting coping, self controlling and planful problem solving are examples of problem-focused coping ways and emotion-focused coping strategies consist of self blame, escape avoidance and distancing. Seeking social support is a combined way that deals with stress using both types of strategies (6).

Furthermore, based on different studies, mental health and wellbeing are fundamental to quality of life (7) and using adaptive coping strategies can improve quality of life (8). On the other hand, it is clear that the presence of positive mental health or ‘wellbeing’ influences health outcome across a wide range domains and one of the most important area in this pathway is improved quality of life (9). In 2000, Grey and colleagues found that training the adaptive coping mechanisms to diabetic patients could be improved the quality of life and controlled the metabolic changes (10). In Iran, in 2006, Taleghani conducted a project related to coping mechanisms and quality of life in women with breast cancer. The results showed that most of patients coped with stress using emotion focused coping strategies and the religion was important for them (11). Review literature represents that few studies have been conducted about coping mechanisms in community level in Iran. It has only included a case study of Isfahan Healthy Heart Project which investigated the relationship between ways of coping and lifestyle (12).

## 2. Objectives

In this study, we intended to evaluate the coping mechanisms in women at community level and determine its relationship with demographic factors and quality of life domains. In this descriptive study as a part of interventional community based research in promoting the community mental health, has been attempted to explain the situation of target group in pre interventional stage.

## 3. Patients and Methods

This is a descriptive cross sectional study that was implemented in 2012 in one of the western part of Tehran (district 22) in Iran. The reason for the chosen area is the presence of volunteers to participate in community based interventional programs and the active nongovernmental organizations (NGOs) evident in this area. Also, data gathering was implemented by volunteers. They were chosen according to the following criteria: Iranian married women 25-65 years who residents in district 22, having at least high school diploma and their motivation and interest in participatory activities. After interviewing with 20 candidates, 10 of them were selected. The target group was married adult women. The inclusion criteria for them included:

1-Iranian married women aged 18-65 who reside in the defined district.

2-Willingness to participate in the research.

All singles and also married women below 18 and above 65 years were excluded from this study. District 22 of Tehran municipality has 30782 households and 7 zones. The average number of households in each zone is almost 4500.There is all of household information records in health centers. For conducting the study, two zones were selected, control and intervention zones. The list of target group based on inclusion criteria was provided for each zone. The sample strategy is simple random sampling. In this method, each person has equal chance to participate in the study. In each zone (control and intervention zones), 100 married women were selected. This sample was sufficient enough to estimate an indicator of quality with maximum prevalence of 50%, 95% confidence and 10% accuracy. The expected power in this study is 80%. All of the data were gathered by trained volunteers via home visits and face to face relationship. In order to provide privacy, the questionnaire was filled out by trained volunteer lonely. In our study missing data was a few and ignorable.

### 3.1. Questionnaire Measures

#### 3.1.1 Way of Coping (WOC) Questionnaire by the Folkman and Lazarus

The tool a 66-item, self-report questionnaire using a four-point Likert scale that can be completed in approximately twenty minutes. This has been designed to assess coping processes in response to a specific stressful event experienced by the sample during the past four weeks. This tool has been designed to assess the eight ways of coping include: Confrontive Coping, Distancing, Self Controlling, Escape–Avoidance (emotion-focused coping) and Seeking Social Support, Accepting Responsibility, Planful Problem Solving and Positive Reappraisal( problem-focused coping) . Individuals responded to each item on a four-point Likert scale ranging from 0 (does not apply and/or not used) to 3 (used a great deal) showing the frequency of each strategy which were used. The construct reliability of farsi version of WOC was found 0.6 -0.84 by padyab in 2006(13). Alpha coefficient in this study was 0.83 which indicated adequate internal consistency and reliability.

#### 3.1.2 Quality of Life Questionnaire (WHO, QOL-BREF)

This tool consists of 26-items on a five point Likert scale. This tool has four domains as mental, social, environmental and physical health. This questionnaire has been translated in more than 15 countries in the world such as Iran. An Iranian reliability study showed that Cronbach's alphas for the four domains of the WHO QOL-BREF were satisfactory (physical health=0.81, psychological status=0.78, social relationships=0.82, and environmental conditions=0.80(14).

The domain score is converted to a transformed score (ranging from 4 to 20) to enable comparison between domains. A higher score denotes a higher QOL. The domain scores were computed on the basis of WHO profiles(15).

### 3.2. Data Analysis

The collected data were analyzed using the SPSS version 11 statistical software packages. Independent T test, Mann-Whitney U and other descriptive tests has been considered to analyze the data.

### 3.3. Ethical Consideration

All of ethical considerations were considered in this study. Before completing the questionnaire, all participants were assured about the confidentiality of their responses and anonymity of their participation and verbal informed consent was obtained. This study was approved in 2012 by the Ethics Committee of University of social welfare and rehabilitation science.

## 4. Results

In this study, 200 married women participated totally. The mean age of the participants was 41 (SD = 10), (Min age = 21 and Max age = 65 years). More than 90 percent of women lived with their spouses and only 2 percent of them were divorced at the stage of the research. The average duration of marriage was 17.3(SD = 12) and at least 50 percent of the participants lived with their husbands more than 15 years (Min = 1 and Max = 50). The average number of children in this study was 2 (Min = 0 and Max = 6). Considering the educational status, 55 percent of participants had a diploma or less, 35 percent were bachelor and the reminder had higher level. Fifty five percent of the participants were housewives. Table 1 shows the demographic characteristic in control and intervention groups. 

**Table 1.** Demographic Characteristic in Control and Intervention Zones

Demographic Characteristic	Control, Mean (SD)	Intervention, Mean (SD)	Significance Level
**Age**	40.35 (9.7)	41.5 (10.4)	N.S ^a^
**Duration of marriage**	16.25 (11.8)	18.32 (12.3)	N.S
**Marital status**			
Married	93%	92%	N.S
Divorced/ Death of spouse	7%	8%	N.S
**Education**			
Non-academic education	56%	54%	N.S
Academic education	44%	46%	N.S
**Employment status**			
Employed	44%	45%	N.S
housewives	56%	55%	N.S

^a^ N.S, No Significance.

Whereas there is no significant difference in demographic characteristics between control and intervention groups, after this, we will consider both of them as one group (n = 200). Based on the folkman and Lazarus theory in coping strategies, there are eight ways in dealing with stress. In this study, planful problem solving is the most preferred way of coping that married women use dealing with stress. The least way is confrontive coping. It means that the target group in 5% of cases uses confrontive coping way to stress dealing. Table 2 shows the percent of using different ways of coping in target group. The range of this score is 0-100. 

**Table 2.** Ways of Coping Relative Scoring in Married Women in Western Part of Tehran

Way of coping	Relative scoring ^a^	Standard Deviation (SD)
**Planful Problem Solving (PPS)**	0.19	0.04
**Positive Reappraisal(PR)**	0.18	0.03
**Escape – Avoidance (EA)**	0.15	0.04
**Seeking Social Support(SSS)**	0.13	0.04
**Accepting Responsibility(AR)**	0.12	0.04
**Distancing(D)**	0.11	0.04
**Self Controlling(SC)**	0.07	0.04
**Confrontive Coping(CC)**	0.05	0.06

^a^ The Average Score for each Scale is Divided by the Sum of the Averages for all 8 Scales

For illustrating the association between ways of coping and demographic characteristics, at first, the normal assumption of numerical variables was checked by Kolmogorov Smirnov test. In related to different ways of coping, escape-avoidance and distancing have normal distribution and for others, this assumption isn’t true. Thus, for these two ways, we use independent samples T test and for others, Mann-whitney U was used.

**Table 3.** Association Ways of Coping and Demographic Characteristics

Way of Coping	Employment Status	Educational Level	Marital Status
**Planful Problem Solving (PPS)**	N.S ^a^	N.S	N.S
**Positive Reappraisal(PR)**	N.S	N.S	N.S
**Escape – Avoidance (EA)**	N.S	S (P < 0.001)	N.S
**Seeking Social Support(SSS)**	N.S	N.S	N.S
**Accepting Responsibility(AR)**	N.S	N.S	N.S
**Distancing(D)**	N.S	S (P < 0.001)	N.S
**Self Controlling(SC)**	N.S	N.S	N.S
**Confrontive Coping(CC)**	N.S	S	N.S

^a^No Significant; P > 0.050

According to results, there was no significant difference between ways of coping and women occupation (P > 0.050). There was a significant difference between Escape-Avoidance, Distancing and Confrontive Coping ways in different levels of education. It means that using of these ways in women with academic education were less than others (P < 0.001). But others haven’t significant difference with educational levels. Bivariate correlations indicated no significant difference between duration of marriage and ways of coping. Number of children had positive significant correlation with Distancing way (P < 0.005). It means that, mothers who have more children use Distancing way more than others in dealing with stress. The quality of life questionnaire has six parts. The first two related to quality of life and health satisfaction as generally and assess each by one question. In this study, the mean score of quality of life and health satisfaction were 15.2 (SD = 3.05) and 14.68 (SD = 3.54). The score of main domains provided in Quality of Life questionnaire has been presented in Table 4. The range of domains score is 4-20. 

**Table 4.** The Score of Main Domains of WHO, QOL-BREF in Target Group

Domain	Mean	SD
**Physical**	14.16	2.61
**Mental**	13.36	3.02
**Social**	14.42	3.2
**Environmental**	14.27	2.53

Table 4 indicates that the mental domains had minimum mean score. Table 5 shows the association between quality of life domains and demographic characteristics. Quality of life, health satisfaction and social domain have normal distribution and for others, the normal assumption isn’t true and Mann –Whitney U (Non parametric test) was used for analysis. 

**Table 5.** Association between Quality of Life Domains and Demographic Characteristics

Main Domains of WHO, QOL-BREF	Employment Status	Educational Level	Marital Status
**Quality of life**	N.S ^a^	S ^b^	S
**Health satisfaction **	S	S	S
**Physical domain**	S	S	S
**Mental domain**	N.S	S	S
**Social domain**	S	S	S
**Environmental domain **	N.S	S	N.S

^a^ no significant: P > 0.05

^b^ significant: P < 0.05

The results show that the quality of life has negative significant correlation with age and also physical domain has negative significant correlation with age and marriage duration (P < 0.001).

**Table 6.** Mean Scores of Quality of Life Domains Based on Women Aged Groups

Age group	Physical Health Mean (SD)	Psychological Health Mean (SD)	Social Relationship Mean (SD)	Environment Mean (SD)
**21-30**	15.28 (1.6)	13.93 (2.3)	14.57 (2.8)	14.40 (1.8)
**31-40**	14.44 (2.4)	13.71 (2.9)	14.68 (2.8)	14.38 (2.5)
**41-50**	13.74 (2.9)	12.75 (3.5)	14.22 (3.8)	14.07 (2.9)
**51-60**	13.82 (2.2)	13.37 (2.7)	14.79 (2.5)	14.35 (2.5)
**Above 60**	10.85 (2.8)	12 (1.8)	10.44 (4.1)	13.91 (1.1)
**Total**	14.16 (2.6)	13.36 (3.02)	14.42 (3.2)	14.27 (2.5)

Table 6 illustrates mean scores of quality of life domains based on women aged groups. The results show that in older ages, the quality of life reduces. There was a significant positive correlation between quality of life and using Positive Reappraisal and Planful Problem Solving ways. This correlation was negative in using Confrontive Coping and Escape – Avoidance ways with quality of life domains (P < 0.05). It means that, more using focus based coping strategies can increase the mean score of quality of life. 

## 5. Discussion

This study investigated coping style of married women who live in western part of Tehran. Results showed that ways of coping have significant relationship with education. Also study of Huizink in 2002 indicated that education has the positive role on using problem oriented coping in women (16). The results (16) showed that mothers with more children use more distancing and self controlling ways. Different studies have indicated that these ways of emotion based coping increase distress (17). Review of our results represented that married women reported problem focus coping in less than half of the participations. Different studies suggest that women likely use more strategies which involve verbal expressions to others, seek emotional support and ruminate about problems (18).

In another study in Shiraz University of Medical Sciences, nurses used Confrontive Coping and Positive Reappraisal ways in dealing with stress. One of the reasons in using more Positive Reappraisal is the relationship of this way with religious dimensions, and Iranian people apply more religious coping ways than other countries. It can be the effect of spirituality role in stress coping. In addition, both Planful Problem Solving and Positive Reappraisal are coping methods that can be enhanced through educational preparation and work experience (19). In this study, although there was no significant difference between way of coping and women occupation, there was a relationship between quality of life and employment. The study of Shahmiri on ways of coping in depressed patients showed that using Confrontive Coping and Planful Problem Solving methods in employees are more than housewives. These ways are known as adaptive problem-focus coping strategies that decrease distress.

Regarding to participants quality of life, the results showed that social relationship had the maximum score and mental health domain had the minimum score. The study of Nejat and her colleagues related to women quality of life in Tehran in 2007 indicated that physical health had maximum and environmental domain had minimum scores but the mean score of mental health was almost the same in both studies (14). The study of Agnihotri and colleagues (20) in 2010 in India represented that the social relations had the maximum score and environmental domain had the minimum. The mean score of mental health in that country was 72.9 (in 0-100 scale). Based on different studies, Quality of Life assessment is useful to develop interventional strategies and evaluation outcomes of these interventions at individual and community level.

### 5.1. Limitation

The results of this study must be interpreted with considering to some methodological limitations. Our results relied on self-report entirely of married women. This study also used a cross-sectional design; therefore it’s not possible to draw causal inferences. On the other hand the community based participatory research is influenced by social trust and it may be fluctuated over the time.

### 5.2. Strong Point

The community based participatory research is an effective method to mental health promotion. Participation of volunteers in this project strengthens the social support network and it is necessary for community empowerment. Missing data in this study is the least. No confounding variables were detected.

Implementing an appropriate interventional program considering the adaptive ways of coping with stress is effective in women mental health and quality of life promotion.
